# Bone Mineral Density as a Marker of Cumulative Estrogen Exposure in Psychotic Disorder: A 3 Year Follow-Up Study

**DOI:** 10.1371/journal.pone.0136320

**Published:** 2015-08-26

**Authors:** Christine van der Leeuw, Sanne Peeters, Patrick Domen, Marinus van Kroonenburgh, Jim van Os, Machteld Marcelis, Genetic Risk and Outcome in Psychosis (G.R.O.U.P.)

**Affiliations:** 1 Department of Psychiatry and Neuropsychology, School for Mental Health and Neuroscience, EURON, Maastricht University Medical Centre, Maastricht, the Netherlands; 2 Department of Radiology and Nuclear Medicine, Maastricht University Medical Centre, Maastricht, the Netherlands; 3 Department of Psychosis Studies, Institute of Psychiatry, King’s College London, London, United Kingdom; 4 Institute for Mental Health Care Eindhoven (GGzE), Eindhoven, the Netherlands; The University of Queensland, AUSTRALIA

## Abstract

Altered estrogen-induced neuroprotection has been implicated in the etiology of psychotic disorders. Using bone mineral density as a marker of lifetime estrogen exposure, a longitudinal family study was conducted to discriminate between etiological mechanisms and secondary effects of disease and treatment. Dual X-ray absorptiometry scans were acquired twice, with an interval of 3 years, in 30 patients with psychotic disorder (male (M)/female (F): 24/6, mean age of 32 years at second measurement), 44 non-psychotic siblings of patients with a psychotic disorder (M/F: 26/18, mean age 32) and 27 controls (M/F: 7/20, mean age 35). Total bone mineral density, Z-scores and T-scores were measured in the lumbar spine and proximal femur. Associations between group and bone mineral density changes were investigated with multilevel random regression analyses. The effect of prolactin-raising antipsychotic medication was evaluated. (Increased risk of) psychotic disorder was not associated with disproportionate bone mineral density loss over a three year period. Instead, femoral bone mineral density measures appeared to decrease less in the patient versus control comparison (total BMD: B = 0.026, 95% CI 0.002 to 0.050, p = 0.037; Z-score: B = 0.224, 95% CI 0.035 to 0.412, p = 0.020; and T-score: B = 0.193, 95% CI 0.003 to 0.382, p = 0.046). Current or past use of a prolactin-raising antipsychotic medication was not associated with bone mineral density changes. In this small longitudinal study, there was no evidence of ongoing estrogen deficiency in psychotic disorder as there was no excessive loss of bone mineral density over a 3-year period in patients using antipsychotic medication.

## Introduction

A recent review concluded that “schizophrenia is associated with reduced bone mineral density (BMD) and fracture risk” [1], possibly resulting from a high prevalence of risk factors for osteoporosis in this population [2]. Another recent review reported that BMD is reduced in patients who use AP medication [3], with first generation and other prolactin-raising APs particularly implicated [4–9]. Stubbs and colleagues conducted the first meta-analysis examining low bone mass in schizophrenia and concluded that patients with schizophrenia were more than twice as likely to have osteoporosis in comparison to age and sex matched controls [10].

Interestingly, schizophrenia has been proposed as an independent determinant of skeletal status, irrespective of AP use and other secondary factors [11]. Estrogen is an important factor in bone metabolism, demonstrated by the fact that osteoporosis risk rises after menopause. Taking this a step further, BMD may be an intermediate marker of cumulative estrogen exposure [12] and may reflect alterations in the neuroprotective estrogenic impact in the brain [13]. Maric and colleagues demonstrated that female patients, presenting with a first episode of psychosis, had amassed significantly less bone mass than healthy controls. This may be indicative of a primary role for estrogen in the etiology of schizophrenia, as the confounding influence of medication and lifestyle factors is presumed to be minimal at such an early stage of disease. Theoretically, the impact of estrogen on both brain and bone are not specific to women: androgens are aromatized focally and reach biologically effective concentrations at these tissue sites in men [14].

Previously, we assessed BMD as a marker of lifetime estrogen exposure, in relation to background genetic risk of psychotic disorder [15]. BMD was unaltered in non-psychotic siblings, though it was reduced in the femur of female, but not male, patients with psychotic disorder. While the use of prolactin-raising AP could not be dismissed as a possible contributing factor, there was some evidence to suggest that reduced BMD in female patients represents diminished estrogen-induced neuroprotection, in support of the estrogen hypothesis of schizophrenia. Examining associations between BMD and neuroimaging data obtained in the same group of female patients enabled us to further examine this finding. There was evidence for a positive association between BMD and cerebral cortical thickness (CT) [16], suggesting that low cumulative estrogen levels may be accompanied by reduced neuroprotection.

It remains unclear whether hypoestrogenism is a cause and / or result of psychotic disorder [17]. It is also unclear how hyperprolactinemia causes reduced BMD. Both indirect (in the presence of hypogonadism) and direct pathways (in the absence of hypogonadism) have been proposed [3]. Although four longitudinal studies have been performed examining BMD alterations in relation to prolactin-raising AP [7, 9, 18, 19], prospective studies designed to discriminate between etiological mechanisms and secondary effects of disease and treatment have not been conducted.

After a follow-up period of 3 years, we reassessed BMD in the original sample [15]. We hypothesized that patients would show a disproportionate decline in BMD compared to siblings and controls, reflecting primary factors associated with the disorder (continuous low estrogen levels). Excessive BMD loss in patients and siblings compared to patients would lend support to hypoestrogenism as an endophenotype of psychotic disorder, not influenced by disease-related factors. Though no evidence of reduced BMD in siblings was found at baseline, we wished to confirm this at follow-up. Furthermore, BMD changes in siblings would be of particular interest if siblings transitioned to the patient group during the three year interval. As depression potentially may lead to bone loss [20] and some siblings and controls had a history of major depressive disorder (MDD), this was dealt with in the analyses.

Potential adverse disease-related effects were monitored in patients (including lifestyle factors and AP use), as they may also contribute to reduced BMD [1].

## Materials and Methods

### Participants

Dual X-ray absorptiometry (DEXA) scans were conducted in the context of a longitudinal Dutch study Genetic Risk and Outcome of Psychosis (GROUP) [15, 21]. The study population consisted of patients with a diagnosis of non-organic, non-affective psychosis, their non-psychotic siblings and a control group. Diagnosis was based on DSM-IV criteria [22], assessed with the Comprehensive Assessment of Symptoms and History (CASH) interview [23]. The CASH was also used to confirm the absence of a diagnosis of non-affective psychosis in the siblings, and absence of a diagnosis of any psychotic disorder in the control participants. For the control participants, the occurrence of any psychotic disorder in either the participant or any first-degree family member, assessed using the Family Interview for Genetic Studies, constituted an exclusion criterion. Exclusion criteria included: 1) metabolic or endocrine disease, 2) dietary deficiency or eating disorder, 3) medication use: corticosteroids, thyroxin, anti-epileptic drugs, heparin, lithium, cytostatic agents, 4) (semi-) professional athletes, 5) polydipsia, 6) pregnancy, and 7) hormonal (infertility) treatment. Three years after baseline assessment (T0), all participants were invited to undergo a second DEXA scan (T1).

Forty-three percent (n = 76) of the original sample at T0 was lost to follow-up at T1. Participants either met exclusion criteria at T1 (n = 19, most common exclusion criteria were use of corticosteroids and pregnancy) or they declined further participation for personal or practical reasons. We were unable to contact 14 of the original participants, 11 of whom were patients.

At T1, 30 patients with a psychotic disorder, 44 non-psychotic siblings and 27 controls remained. Twenty-three patients had a diagnosis of schizophrenia, 4 patients had a diagnosis of schizoaffective disorder and 3 patients had a diagnosis of psychotic disorder not otherwise specified. No controls or siblings transitioned to the patient group. Ten controls and 12 siblings had a current or past diagnosis of major depressive disorder (MDD), of whom four controls and two siblings transitioned from no diagnosis to MDD between T0 and T1.

The sample included 14 families. Ten families contributed one patient and one sibling and one family contributed one patient and two siblings. Two families contributed two siblings but no patients. In the control group, one family contributed two siblings. Nineteen independent patients, 28 independent siblings and 25 independent controls participated.

There were also participants at T1 who did not undergo a DEXA scan at T0. This additional group included 24 controls, 15 siblings and 12 patients. Thus, the total sample size at T1 was n = 152.

### Measures

#### Substance use

Substance use was assessed using the Composite International Diagnostic Interview (CIDI) [24]. Cannabis use was assessed as reported frequency of use in the past year. Other drug use was assessed in the same way. Alcohol use was defined as the average number of weekly instances of consumption during the previous 12 months. Tobacco use was defined as the number of daily occasions of use.

#### Physical activity and sunlight exposure

Physical activity and sunlight exposure were expressed in total minutes per week at T1. To quantify physical activity, the total amount of time spent on commuting by foot or bicycle, physical activity at work or school, household chores, active hobbies and sports was summed. Sunlight exposure was calculated by multiplying the number of days per week a person went outside by the average number of minutes spent outside (during daylight) on those days.

#### Use of antipsychotic medication

Current AP use and AP use during the follow-up period were classified by AP type: prolactin (PRL)-raising, i.e. first generation APs, and risperidone, paliperidone and amisulpride; or PRL-sparing, i.e. second and third generation APs with the exception of risperidone, paliperidone and amisulpride. Previous AP use was assessed retrospectively by self-report. Best estimate cumulative AP exposure was determined by multiplying the number of days of AP use with the daily AP dose converted to haloperidol equivalents (in milligrams), and summing all periods of use. Exposure during the 3 year follow-up period and lifetime were calculated for all APs and separately for prolactin-raising APs.

#### Use of contraceptive drugs (exogenous estrogen exposure)

Exogenous estrogen exposure was determined by multiplying the daily dose expressed in micrograms with the total days of use. Exposure was determined for the three year interval between T0 and T1 as well as lifetime exposure up to T1.

#### Age at menarche and dysmenorrhea

Age at menarche and the occurrence of menstrual irregularity were assessed in women.

#### Familial osteoporosis

The occurrence of familial osteoporosis was documented.

### Dual X-ray absorptiometry acquisition and processing

Dual X-ray absorptiometry (DEXA) scans were acquired at Maastricht University Medical Centre with a Hologic Discovery A (Tromp Medical, Castricum, the Netherlands) (NHANES and Ethnic Reference Data) at both T0 and T1. The same procedures for DEXA acquisition were used at both measurements. There were no updates in the machinery or software during the interval. DEXA scans of two anatomical areas were performed: the lumbar spine, vertebrae L2 through L4; and the proximal left femur, specifically the collum, trochanter major, intertrochanteric area and Ward’s triangle. BMD was expressed in grams per square centimeter (g/cm^2^), Z-scores and T-scores. The Z-score compares an individual’s BMD with the mean BMD of a comparable population (with respect to gender, age and ethnicity). The T-score compares an individual’s BMD to peak bone mass (PBM). Peak bone mass is the highest BMD an individual is expected to acquire during life. The T-score is used to diagnose osteopenia and osteoporosis. The World Health Organization employs the following criteria: T-scores in osteopenia lie between -1.0 and -2.5; T-scores in osteoporosis are equal or less than -2.5.

At T1 we encountered an unanticipated complication. Pediatric reference values were applied to participants under the age of 20 at T0. These reference values were apparently more stringent than the references employed for adults. For instance, a female control was age 18 at T0 and the pediatric reference values were used. Though her total femoral BMD declined slightly over the three year follow-up (0.853 g/cm^2^ at T0 and 0.845 g/cm^2^ at T1), Z- and T-scores appeared to improve using adult reference values at age 21 (Z: score -1.2 at T0 and -0.8 at T1; and T-score: -1.3 at T0 and -0.8 at T1).

The present longitudinal study included 4 controls, 6 siblings and 1 patient that had not reached age 20 at T0. Inclusion of these individuals would result in skewed lumbar and femoral delta Z- and T-scores, potentially cancelling out or creating differences between groups. Therefore, these 11 participants were excluded from the Z- and T-score analyses. For the total BMD analyses, we did include these individuals as the absolute BMD is not influenced by age-related reference values.

### Ethics statement

The study was approved by the standing ethics committee of Maastricht University Medical Centre and all participants gave written informed consent in accordance with the committee’s guidelines.

### Statistical analyses

Group differences in BMD measures at T1 (n = 152) and BMD change between T0 and T1 (total BMD lumbar spine and femur n = 101, Z- and T-scores n = 90) were analyzed separately for the lumbar spine and proximal femur. The change (delta, Δ) in lumbar and femoral total BMD, Z-scores and T-scores was calculated as T1 minus T0. Multilevel random regression models were fitted [25] given hierarchical clustering occasioned by the fact that participants were clustered in families, compromising statistical independence of the observations. This was done using the XTREG command in STATA (STATA corp, version 13). BMD measures were the dependent variables in the analyses and group (entered as a linear and dummy variable (controls = 0, siblings = 1 and patients = 2)) was the independent variable. Analyses were adjusted for the a priori hypothesized confounders: gender, age at T1, BMI at T1, exogenous estrogen exposure between T0 and T1, physical activity, sunlight exposure, tobacco and alcohol use, exposure to cannabis and the number of days between T0 and T1. Analyses were repeated with lifetime estrogen exposure replacing 3 year estrogen exposure as a covariate. Results were Simes corrected.

Despite the gender-specific findings in our original study [15], we did not conduct analysis stratified by sex due to low numbers (only 7 men in the control group and 6 women in the patient group).

Planned sensitivity analyses were performed excluding individuals with a history of affective disorder (MDD) in the control and sibling groups because these individuals may be at risk of decreased BMD due to similar factors as in psychotic disorder, such as inactivity, diminished sunlight exposure and medication side-effects.

Main effect of the type of AP use (at T1 and during 3 year interval) on Δ BMD was examined by comparing PRL-raising AP to PRL-sparing and no AP (the latter two combined into one group) using multiple regression procedures; with sex, age and BMI as confounders. Accordingly, the main effect of cumulative exposure to PRL-raising AP (over the 3 year interval and lifetime) on Δ BMD was assessed. Sensitivity analyses were repeated with the further exclusion of patients who used a PRL-raising AP.

## Results

The descriptive characteristics apply to the longitudinal sample. The findings for the cross-sectional sample at T1 (n = 152) and the longitudinal sample are presented separately.

### Descriptive characteristics

There were more female participants in the control group and more males in the patient group. Controls were older than patients and siblings. Patients used more tobacco, cannabis and other drugs than controls and siblings. Siblings used more cannabis than controls. Among women, patients experienced more menstrual irregularities than siblings and controls (see Table 1). Out of 30 patients, 26 currently used AP medication, of whom 14 were prescribed a PRL-sparing agent and 12 were prescribed PRL-raising agents (1 patient used haloperidol, 8 risperidone, 1 paliperidone and 1 amisulpride; one patient used a combination of risperidone and aripiprazole). Of the 4 patients who were AP-free at T1, two were AP-free during the entire 3 year interval, and 2 used a PRL-sparing AP (1 aripiprazole, 1 olanzapine) between T0 and T1. The 14 patients with a PRL-sparing AP at T1 did not use a PRL-raising AP at any time during the 3 year interval. However, with the exception of 4 individuals, all patients did use a PRL-raising AP at some point during their lives. Cumulative AP exposure is listed in Table 1. Several patients used other classes of psychotropic medication: eight used antidepressants (of whom 7 a selective serotonin reuptake inhibitor (SSRI) or serotonin and noradrenalin reuptake inhibitor (SNRI), and 1 trazodone), three used benzodiazepines and there was one instance of stimulant (methylphenidate) use. One sibling used mirtazapine and one control used paroxetine, both had a history of MDD. One sibling had briefly used a benzodiazepine due to anxiety (no diagnosis was made), and one sibling and one control had briefly used diazepam due to back pain.

**Table 1:** Demographics.

	Controls, n = 27	Siblings, n = 44	Patients, n = 30
**Sex (n), male / female**	7 / 20	26 / 18	24 / 6
**Age at scan**	35.22 ± 11.60	32.32 ± 7.97	32.03 ± 6.04
**Body Mass Index (BMI) (kg per m** ^**2**^ **)**	25.24 ± 4.49	24.02 ± 3.94	25.35 ± 4.47
**Age at menarche (years)**	13.35 ± 1.50	12.67 ± 1.24	13.17 ± 1.17
**Dysmenorrhea (n)**	9	6	5
**3 year exogenous estrogen exposure (μg)**	5691 ± 8057	8995 ± 10882	9551 ± 11072
**Lifetime exogenous estrogen exposure (μg)**	34542 ± 27201	46674 ± 48460	25090 ± 42206
**Smoking (cigarettes per day)**	1.52 ± 3.61	2.61 ± 6.39	12.43 ± 10.71
**Alcohol consumption (units per week)**	7.23 ± 13.74	6.75 ± 7.16	6.17 ± 9.22
**Cannabis use (number of times past year)**	0.30 ± 1.03	8.75 ± 54.88	66.83 ± 138.69
**Other drug use (number of times past year)**	0	0.05 ± 0.30	3.47 ± 10.01
**Activity (minutes per week)**	2242 ± 1299	1601 ± 1146	1471 ± 1173
**Sunlight exposure (minutes per week)**	945 ± 565	1084 ± 820	955 ± 868
**Current AP use (n, patients)**			26
**PRL-raising / PRL-sparing AP (n)**			12 / 14
**Current AP dose (hal. eq.)**			4.49 ± 3.38
**3 year PRL-raising AP exposure (hal. eq.)**			1711 ± 2609
**Lifetime PRL-raising AP exposure (hal. eq.)**			5998 ± 7117
**3 year all AP exposure (hal. eq.)**			4899 ± 3933
**Lifetime all AP exposure (hal. eq.)**			12375 ± 10069

Means ± SDs reported. AP: antipsychotic medication; PRL: prolactin; hal. eq.: haloperidol equivalents in milligrams.

### Associations between group and BMD in the lumbar spine and femur

#### Cross-sectional analyses

There were no statistical associations between group (linear trend and dummy variables) and BMD measures at T1 (see Table 2). Although not significantly so, all B’s were positive, including the patient-control and patient-siblings comparisons.

**Table 2:** Cross-sectional associations between group and BMD measures at T1.

	Linear trend	S vs. C	P vs. C	P vs. S
***Lumbar spine***				
**Total BMD**	0.029 (0.081)	0.042 (0.069)	0.051 (0.118)	0.009 (0.757)
**Z-score**	0.199 (0.188)	0.270 (0.199)	0.359 (0.229)	0.089 (0.746)
**T-score**	0.273 (0.072)	0.394 (0.061)	0.481 (0.108)	0.087 (0.752)
***Femur***				
**Total BMD**	0.009 (0.535)	0.005 (0.817)	0.021 (0.481)	0.016 (0.558)
**Z-score**	0.044 (0.688)	0.014 (0.929)	0.104 (0.631)	0.091 (0.651)
**T-score**	0.044 (0.686)	0.025 (0.870)	0.098 (0.650)	0.073 (0.714)

B’s (and p-values) reported. B represents the regression coefficient of the multilevel regression analyses. S vs. C: siblings vs. controls; P vs. C: patients vs. controls; P vs. S: patients vs. siblings.

#### Longitudinal analyses

**Fig 1:**
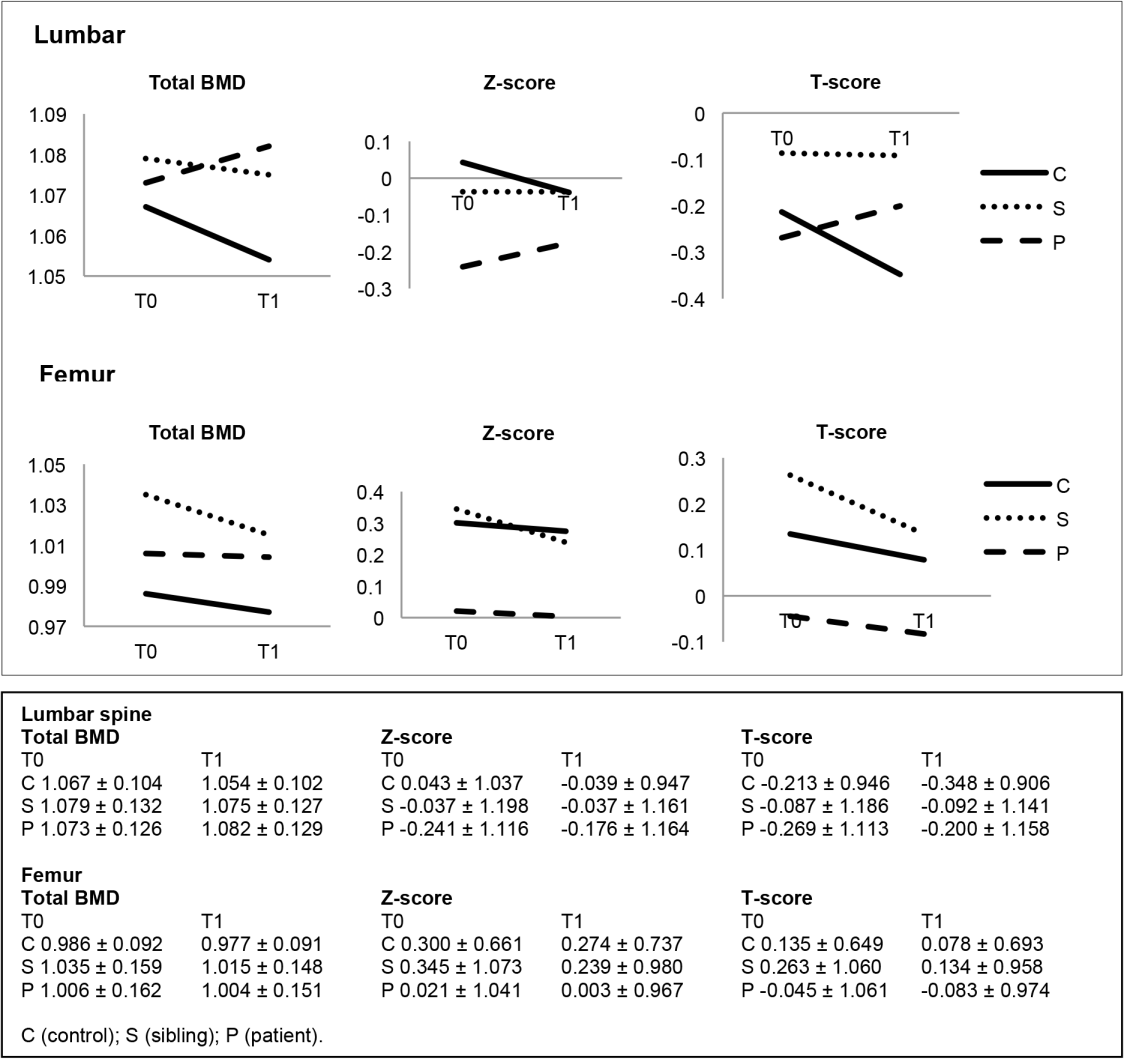
Mean BMD measures at T0 and T1 in the longitudinal sample.

Mean BMD measures at T0 and T1 are depicted in Fig 1. Only patients showed incremental mean BMD change, specific to the lumbar spine. The longitudinal associations between group and BMD change are stated in Table 3. Depending on the direction of mean BMD change, a positive association in the group comparisons signifies either an increase or smaller decrease in BMD measures. There was a significant positive association between group (linear trend) and the Δ Z-score of the femur (B = 0.111, 95% confidence interval (CI) 0.008 to 0.215, p = 0.035). The association for the Δ total femoral BMD and Δ T-score were positive at trend-level significance (see Table 3).

**Table 3:** Longitudinal associations between group and Δ BMD measures.

	Linear trend	S vs. C	P vs. C	P vs. S
***Lumbar spine***				
**Total BMD**	-0.002 (0.813)	-0.002 (0.802)	-0.003 (0.812)	-0.001 (0.954)
**Z-score**	0.003 (0.963)	0.013 (0.910)	0.006 (0.965)	-0.006 (0.956)
**T-score**	-0.002 (0.979)	0.020 (0.856)	-0.006 (0.966)	-0.026 (0.824)
***Femur***				
**Total BMD**	0.012 (0.063)	0.008 (0.381)	0.026 (0.037)*	0.018 (0.092)
**Z-score**	0.111 (0.035)*	0.100 (0.195)	0.224 (0.020)*	0.123 (0.130)
**T-score**	0.097 (0.068)	0.101 (0.195)	0.193 (0.046)*	0.092 (0.262)

B’s (and p-values) reported. B represents the regression coefficient of the multilevel regression analyses.

*p-value <0.05.

For the femur, there were positive associations between group and Δ BMD measures in the patient-control comparison (total BMD: B = 0.026, 95% CI 0.002 to 0.050, p = 0.037; Z-score: B = 0.224, 95% CI 0.035 to 0.412, p = 0.020; and T-score: B = 0.193, 95% CI 0.003 to 0.382, p = 0.046). For the lumbar spine, there were no significant findings in any of the group comparisons (see Table 3).

The use of lifetime estrogen exposure (instead of 3 year estrogen exposure) as a covariate in the analyses led to additional findings. The association between group (linear trend) and Δ femoral BMD measures became more apparent (total BMD: B = 0.015, 95% CI 0.001 to 0.029, p = 0.032; Z-score: B = 0.139, 95% CI 0.031 to 0.246, p = 0.012; and T-score: B = 0.123, 95% CI 0.016 to 0.231, p = 0.025). The significant positive associations in Δ femoral BMD values in patients compared to controls was upheld (total BMD: B = 0.032, 95% CI 0.007 to 0.058, p = 0.013; Z-score: B = 0.280, 95% CI 0.085 to 0.475, p = 0.005; and T-score: B = 0.248, 95% CI 0.053 to 0.443, p = 0.013), and Δ total femoral BMD and Z-score in patients compared to siblings became statistically significant (total BMD: B = 0.024, 95% CI 0.002 to 0.045, p = 0.033; Z-score: B = 0.170, 95% CI 0.004 to 0.337, p = 0.045).

For the lumbar spine, there were no significant findings in any of the group comparisons.

The significant findings described above were not upheld after Simes correction.

### Associations between prolactin-raising antipsychotic medication and BMD change

Delta BMD measures were not significantly different in patients who used a PRL-raising AP compared to those who used a PRL-sparing AP or were AP-free (both at the time of DEXA acquisition and during the entire three year follow-up period). Neither the 3 year nor the lifetime cumulative exposure to PRL-raising APs was associated with Δ BMD (results not shown). Exposure to all AP during the 3 year interval had very small statistically significant effects on Δ femoral BMD measures (total BMD: B = 4.93∙10^−6^, 95% CI 1.07∙10^−6^ to 8.79∙10^−6^, p = 0.014; Z-score: B = 2.64∙10^−5^, 95% CI 1.79∙10^−8^ to 5.29∙10^−5^, p = 0.050; T-score B = 3.16∙10^−5^, 95% CI 4.63∙10^−6^ to 5.86∙10^−5^, p = 0.024), that did not survive Simes correction. Lifetime exposure to all AP did not influence BMD change.

### Exclusion of affective disorder and prolactin-raising antipsychotic medication

#### Cross-sectional analyses

Exclusion of siblings and controls with a history of MDD from the analysis did not result in significant findings at T1. Further exclusion of patients who used a PRL-raising AP resulted in an isolated finding of a significant positive association in the patient-sibling comparison for the total femoral BMD (B = 0.076, 95% CI: 0.004 to 0.147, p = 0.038), which did not survive Simes correction.

#### Longitudinal analyses

Exclusion of siblings and controls with a history of MDD from the analysis yielded similar results for the lumbar spine (no Δ BMD differences between groups). As in the main analysis, there was a significant positive association between group (linear trend) and the Δ Z-score of the femur (B = 0.131, 95% CI 0.011 to 0.251, p = 0.033), while the association for total femoral BMD and the T-score at trend-level significance (total BMD: B = 0.015, 95% CI -0.001 to 0.031, p = 0.059; and T score: B = 0.116, 95% CI -0.004 to 0.235, p = 0.058). The positive associations between group and Δ femoral BMD measures in the patient-control comparison remained (total BMD: B = 0.031, 95% CI 0.003 to 0.059, p = 0.030; Z-score: B = 0.260, 95% CI 0.050 to 0.471, p = 0.015; and T-score: B = 0.230, 95% CI 0.019 to 0.440, p = 0.033). In addition, the associations in the patient-sibling comparison became significant (total BMD: B = 0.027, 95% CI 0.003 to 0.051, p = 0.027; Z-score: B = 0.210, 95% CI 0.029 to 0.390, p = 0.023; and T-score: B = 0.179, 95% CI 0.001 to 0.360, p = 0.052). After further exclusion of patients who used a PRL-raising AP from the analyses only the patient-control comparison in femoral BMD changes remained partially significant (total BMD: B = 0.032, 95% CI -0.001 to 0.066, p = 0.058; Z-score: B = 0.269, 95% CI 0.012 to 0.526, p = 0.040; and T-score: B = 0.254, 95% CI 0.001 to 0.508, p = 0.049).

All findings described above were not upheld after Simes correction.

## Discussion

Contrary to our hypothesis, BMD did not decrease disproportionately in patients compared to siblings and controls. The current or past use of a prolactin-raising AP was not found to be negatively associated with Δ BMD measures.

### Findings

The finding of the original study [15] at T0, namely reduced femoral BMD in female patients (as well as a trend in the whole patient group), is consistent with the conclusions of a review [1] and meta-analysis [10], i.e. lower BMD is prevalent in schizophrenia. Replication of the main baseline findings (reduced femoral BMD in female patients) was not possible in the follow-up sample due to considerable sample size reduction and related power loss, preventing the realization of analyses stratified by sex. Despite evident group differences in risk factors such as smoking, no statistical differences between groups were found in the cross-sectional analysis at T1. Similar to the results at T0 (Van der Leeuw et al., 2013a), siblings were not statistically different from controls at T1, confirming that BMD does not appear to be an endophenotype of psychotic disorder.

The lines in the graphs in Fig 1 displaying mean femoral BMD measures at T0 and T1 seem to converge slightly, possibly suggesting a diminished difference at T1. Indeed, our findings do not indicate excessive loss of BMD in patients. Instead, femoral BMD measures appeared to decrease less in the patient versus control comparison (reflected by a significant positive B in the regression analyses), although these findings were not upheld after Simes correction. Controls, with a mean age of 35 and 3 years senior to siblings and patients, showed a drop in all mean lumbar BMD measures and total femoral BMD. This is compatible with normal bone physiology as peak bone mass is retained until around age 30 and then begins to decline. As age is the only moderator of bone mass loss that survives Bonferroni correction in meta-analysis [10], our patients were possibly too young at T1 to show disproportionate BMD loss compared to (slightly older) controls. Matching for gender and age would have enabled a superior comparison. Matching for gender would also have been advantageous to prevent the uneven sex distribution in and between our study groups. There are more men than women in the patient group and disproportionately more women in the control group. In our previous study [15], BMD was lower specifically in female patients. In the present study, BMD decline in all patients (men and women) may be less evident owing to the larger proportion of males in this group, in comparison to the predominantly female control group. This possibility cannot be excluded, as stratified analyses per sex were not deemed feasible in the present study due to insufficient sample size (see below under Methodological considerations).

Notably, patients currently using PRL-raising AP did not show accelerated BMD loss compared to patients taking PRL-sparing AP or no AP. Nor did cumulative three year or lifetime exposure to PRL-raising AP have a main effect on BMD change. Exposure to all AP in the 3 year interval appeared to have a very small positive effect, of which the clinical relevance is questionable.

The interpretation of our findings concerning AP are further complicated by the fact that AP data was largely collected retrospectively by self-report. This is prone to bias, possibly rendering the AP data unreliable. Ideally, the participant supplied an overview of medication history from their doctor or pharmacy. It was standard practice to request this of the participant, though participants did not routinely comply.

The meta-analysis by Stubbs reports that neither PRL-raising AP use nor confirmed hyperprolactinemia contributed to the higher prevalence of low bone mass in schizophrenia; however, they conclude that heterogeneity between studies may underlie the absence of association [10].

Though neither the present study nor the meta-analysis by Stubbs [10] report evidence of adverse effects of PRL-raising AP, it is premature to conclude that such an effect is absent, due to the many methodological issues addressed above. Larger longitudinal studies are warranted.

More optimistically, our findings may be reassuring for patients who take prolactin-raising AP in the short term. In the long run, it deserves recommendation to remain cautious of sequelae of hyperprolactinemia such as hypogonadism and BMD loss, and to assess side-effects of prolactin-raising AP in each individual patient.

### Longitudinal studies

Longitudinal studies investigating BMD in psychotic disorder are scarce. Four other studies have been carried out to date. Recently, in a larger study (163 patients and 90 controls), Wang and colleagues found that hyperprolactinemia resulted in decreased total lumbar BMD in a group of male and female patients with schizophrenia (mean age of 36) who had been treated with PRL-raising APs for 12 months [18]. An observational cohort study [19] examined longitudinal BMD changes in patients, comparing patients taking PRL-raising AP (n = 141) and PRL-sparing AP (n = 23) at first DEXA measurement. The mean follow-up and main finding is comparable to the present study. There was no significant BMD difference between patients taking PRL-raising or PRL-sparing AP at each measurement, over a mean follow-up period of 3.4 years. However, a significant AP type by time interaction was found, possibly indicating a negative effect of prolactin-raising AP. Two studies examined AP effects on BMD specifically in women between the ages of 22 and 53 years (mean 36 years) [9] and at premenopausal age (not specified in years) [7]. Over a period of 12 months, Abraham et al. found that hyperprolactinemia accelerated bone metabolism, but this did not result in bone mass loss [9]. The latter finding (absence of bone mass loss) is consistent with the current findings in patients (although both sexes were included in our study), in whom we did not find significant changes in BMD despite the common use of PRL-raising AP from a lifetime perspective. Meaney and O’Keane [7] followed premenopausal female patients with schizophrenia over the course of one year and compared lumbar and femoral BMD changes between those taking prolactin-sparing AP and those taking prolactin-raising AP. In that study, the efficacy of interventions (such as weight-bearing exercise, nutritional supplements and sex hormone replacement therapy) to ameliorate BMD was examined. Without intervention, women on a PRL-raising AP showed a decrease in lumbar BMD compared to women taking a PRL-sparing AP. With intervention, patients using PRL-sparing AP (n = 4) showed a significant increase in lumbar BMD, while patients using a PRL-raising AP (n = 12) also showed an increase, though not significantly so [7]. No differences in femoral BMD were found, between the AP or intervention groups. Although the incremental change was specific to total femoral BMD in our study, Meaney and O’Keane’s findings in the lumbar spine lend some support to our findings as they show a beneficial evolution of BMD change in a small group of patients using PRL-sparing AP. The majority of our patients used PRL-sparing AP or no AP.

An alternative explanation for the absence of progressive BMD loss in the patient group of the current study is that therapeutic interventions may have occurred to improve BMD over the course of the 3 year follow-up in participants with low BMD. If the DEXA scan at T0 was indicative of osteopenia or osteoporosis, the participant’s general practitioner was alerted and treatment may have been started. As the study design was naturalistic, these interventions were not monitored between T0 and T1 and did not constitute an exclusion criterion at T1.

### Methodological considerations

Our study makes a new contribution to the existing literature, despite some limitations. At T0 study size was moderate. Due to a loss to follow-up of 43%, sample size at T1 was reduced quite drastically. Thus, the longitudinal analyses may have been underpowered and the possibility of false-negative outcomes cannot be excluded. As the main finding of reduced femoral BMD in the original study (T0) was specific to the female patient sample, we wished to reexamine sex differences at T1. Female patient sample size was already marginal at T0 (n = 16), unfortunately this number declined further at T1 (n = 6) and stratified analyses per sex were not deemed feasible. Again, larger longitudinal studies are warranted.

There were large standard deviations of the mean of the estrogen variables (3 year and lifetime exposure). Ideally, correction for this phenomenon would have constituted sensitivity analysis excluding women with a history of exogenous estrogen use. Our study sample had inadequate power for such analyses: only four controls, four siblings and two patients had never used exogenous estrogen. Furthermore, it is questionable if a study of “exogenous estrogen-naïve” women is realistic in our catchment area as hormonal contraceptive use (from a relatively young age) is quite common in the Netherlands.

The present study had an adequate follow-up duration, comparable to only one other study [19] that had a similar mean follow-up with a maximum up to five years. BMD is the product of the continuous process of bone turnover in which bone is both resorbed and newly generated. Bone metabolism is influenced by many factors, including endocrinologic and immunologic status [2]. Three to four years are required before BMD changes can be detected by radiologic examination [26]. Abraham et al. argued that their follow-up of one year may have been insufficient for changes in bone metabolism to result in actual loss of BMD [9]. The three year follow-up in this study did not indicate disproportionate loss of BMD, though markers of bone metabolism were not available to examine bone turnover rates and serum prolactin measurements were lacking. Furthermore, sex hormone and prolactin levels were not assessed. Interpretation of estrogen levels is complicated as its measurement is a momentary assessment of naturally fluctuating concentrations. Given our primary interest in cumulative estrogen exposure, BMD was considered the best outcome measure. Nevertheless, although estrogen and prolactin levels do not represent lifetime exposure, they may have contributed to the understanding of our findings.

Serum vitamin D concentration would have been another useful laboratory measure. It would certainly have been superior to the variable sunlight exposure (in minutes per week spent outside during daylight). Physical activity was also expressed in minutes per week, calculated by a self-report questionnaire conceived for this study. The use of a standardized instrument to measure physical activity would have been desirable. Soundy et al. delineate recommendations in their review [27] for future practice. Self-report questionnaires are subject to recall bias, even more so in studies including participants with cognitive symptoms due to schizophrenia.

The inclusion of siblings and controls with MDD in the study sample is another issue worth addressing. This issue was addressed by correcting for (shared) risk factors between psychotic disorder and MDD in the primary analysis. Furthermore, controls and siblings with MDD were excluded in sensitivity analyses (again to account for these risk factors as well as the sparse use of medication among siblings and controls with MDD as described under 3.1 Descriptive characteristics).

Due to the issue of discrepant pediatric and adult reference values (described under section 2.3), total BMD change (larger sample, n = 101) should serve as a benchmark with which Z- and T-score changes (in smaller sample of 90) should correspond. This was the case for the majority of our findings. Therefore, we feel that we adequately addressed issue of discrepant reference values and our findings were not greatly influenced by this caveat.

A major distinction of the present study is that it is unique in its focus on a possible etiological mechanism, i.e. (persisting) primary low estrogen levels. However, designing a study to elucidate a primary etiological effect for estrogen in psychotic disorder remains challenging. Although BMD was used as a marker of cerebral estrogen exposure, we should bear in mind that different tissue sites have different estrogenic receptors and effects. Furthermore, BMD is influenced by many (non-) hormonal factors. It is, at best, a proxy marker of estrogen-mediated neuroprotection.

### Conclusions

In this small longitudinal study, there was no evidence of ongoing estrogen deficiency in psychotic disorder as there was no excessive BMD loss over a 3-year period in patients using AP. In line with previous cross-sectional work, BMD did not appear to be an endophenotypic marker of psychotic disorder.
